# Hyperthyroidism, but not hypertension, impairs PITX2 expression leading to Wnt-microRNA-ion channel remodeling

**DOI:** 10.1371/journal.pone.0188473

**Published:** 2017-12-01

**Authors:** Estefanía Lozano-Velasco, Rosemary Wangensteen, Andrés Quesada, Carlos Garcia-Padilla, Julia A. Osorio, María Dolores Ruiz-Torres, Amelia Aranega, Diego Franco

**Affiliations:** 1 Cardiac and Skeletal Muscle Development Group, Department of Experimental Biology, University of Jaen, Jaen, Spain; 2 Department of Health Sciences, University of Jaen, Jaen, Spain; University of Kansas Medical Center, UNITED STATES

## Abstract

PITX2 is a homeobox transcription factor involved in embryonic left/right signaling and more recently has been associated to cardiac arrhythmias. Genome wide association studies have pinpointed PITX2 as a major player underlying atrial fibrillation (AF). We have previously described that PITX2 expression is impaired in AF patients. Furthermore, distinct studies demonstrate that Pitx2 insufficiency leads to complex gene regulatory network remodeling, i.e. Wnt>microRNAs, leading to ion channel impairment and thus to arrhythmogenic events in mice. Whereas large body of evidences has been provided in recent years on PITX2 downstream signaling pathways, scarce information is available on upstream pathways influencing PITX2 in the context of AF. Multiple risk factors are associated to the onset of AF, such as e.g. hypertension (HTN), hyperthyroidism (HTD) and redox homeostasis impairment. In this study we have analyzed whether HTN, HTD and/or redox homeostasis impact on PITX2 and its downstream signaling pathways. Using rat models for spontaneous HTN (SHR) and experimentally-induced HTD we have observed that both cardiovascular risk factors lead to severe Pitx2 downregulation. Interesting HTD, but not SHR, leads to up-regulation of Wnt signaling as well as deregulation of multiple microRNAs and ion channels as previously described in Pitx2 insufficiency models. In addition, redox signaling is impaired in HTD but not SHR, in line with similar findings in atrial-specific Pitx2 deficient mice. *In vitro* cell culture analyses using gain- and loss-of-function strategies demonstrate that Pitx2, Zfhx3 and Wnt signaling influence redox homeostasis in cardiomyocytes. Thus, redox homeostasis seems to play a pivotal role in this setting, providing a regulatory feedback loop. Overall these data demonstrate that HTD, but not HTN, can impair Pitx2>>Wnt pathway providing thus a molecular link to AF.

## Introduction

Atrial fibrillation (AF) is the most frequent arrhythmogenic defect in the human population, with an estimate incidence of 2–4% in the general population but rising up to 10% in the elderly [1]. Genetic mutations in a large array of ion channel encoding genes have been described, although only representing <10% of AF cases [2–5]. Recently, genome wide association studies (GWAS) have identified a discrete number of risk variants linked to AF. In particular, SNPs located in chromosome region 4q25, thus in the vicinity of PITX2/ENPEP, display highest association significance [6], while other SNPs linked to ZFHX3 (16q22)[7–8], KCNN3 (1q21) [9] and IL6R (16q13) [10] display more modest significance. Functional evidences demonstrated that 4q25 genomic region containing these risk variants can interact with PITX2 and ENPEP promoter sequences [11]. However it remains elusive how variation within other SNPs (ZFHX3 (16q22), KCNN3 (1q21) and IL6R (16q13)) is mechanistically linked to AF.

Experimental analyses demonstrated that Pitx2 insufficiency leads to atrial arrhythmias [12–14] by modulating distinct ion channels that contribute to the configuration of the cardiac action potential [13–15], as well as cell-cell gap junctional and calcium handling proteins [13,16]. In addition, Pitx2 modulates expression of several GWAS associated genes, such as IL6R, KCNN3 and ZFHX3. Importantly it also regulates WNT8 expression which, in turn, modulates a complex gene regulatory network, including multiple microRNAs, with a large impact on calcium homeostasis control and pro-arrhythmogenic events [16].

It is well-established that the onset of an AF episode triggers subsequent and more severe AF episodes, leading to electrical and structural remodeling of the diseased heart, a condition quoted as “AF begets AF” [17]. Electrical remodeling involves progressive changes in the cardiac electrical properties, leading to early afterdepolarization, delayed afterdepolizations and/or changes in the action potential duration configuration [17], culminating thus in rotor formation [18]. In this context, a pivotal role of reactive oxidative stress has been recently reported [19–20]. Structural remodeling involves atrial dilation, fibrosis and/or inflammation [21] indirectly promoting rotor formation and thus electrical re-entry circuitries [18].

Cardiovascular risk factors such hypertension (HTN), hyperthyroidism (HTD), diabetes and obesity have been repetitively demonstrated to promote onset of atrial fibrillation, respectively [22–24]. Furthermore, the occurrence of AF can be also triggered by preceding cardiovascular diseases such as hypertrophic cardiomyopathy and valvular heart diseases [23,25–26]. Whereas large body of evidences has been provided in recent years on PITX2 downstream signaling pathways, scarce information is available on upstream pathways influencing PITX2 in the context of AF. A seminal study demonstrated that prolonged hypertension is capable of decreasing PITX2 expression [27] but the functional consequences of such changes remain unexplored. Furthermore, PITX2 has been recently reported to control redox homeostasis in skeletal muscle [28] as well as in the regenerating heart [29] but not linked to redox homeostasis and cardiac arrhythmogenic defects has been reported.

In this study we demonstrate that Pitx2 expression is impaired in HTN and HTD experimental models. Interestingly, HTD but not HTN elicits a complex impairment of PITX2>Wnt>microRNA signaling which leads to abnormal ion channel expression. Importantly, ROS signaling is also altered in Pitx2 deficient mice and impaired ROS signaling regulates Pitx2>Wnt>microRNA cascade. Overall, our data demonstrate a complex regulatory network of AF risk factors and PITX2 downstream signaling providing additional molecular insights linking pro-arrhythmogenic substrates and AF.

## Materials & methods

### Experimental models

The Pitx2^floxed^ and NppaCre transgenic mouse lines have been previously described [30–31]. Generation of conditional atrial (NppaCre) mutant mice has been previously described [14–15]. Three different conditions were used for the NppaCrePitx2 mice: wild-type Cre controls (NppaCre2Pitx2^fl/fl^), atrial-specific heterozygous (NppaCre+Pitx2^fl/-^), and atrial-specific homozygous (NppaCre+Pitx2^-/-^). This investigation conforms with the Guide for the Care and Use of Laboratory Animals published by the US National Institutes of Health. The study was approved by the University of Jaén Bioethics Committee.

Male Wistar rats weighing 250–280 g were maintained on standard chow and tap water *ad libitum*. Two groups of animals were analyzed; a) control and b) hyperthyroid rats as previously described [32]. Briefly, hyperthyroidism was induced by injecting s.c. thyroxine (75 Mg/rat/day) for four weeks. Spontaneously hypertensive rats (SHR) and their corresponding Wistar Kyoto controls (WKY) were purchased to Harlan Laboratories with 8 weeks old, and maintained on standard chow and tap water *ad libitum* during 24 weeks. Tail systolic BP (SBP) and heart rate (HR) were recorded once a month by using tail-cuff plethysmography in unanaesthetized rats (LE 5001-Pressure Meter, Letica SA, Barcelona, Spain) as illustrated in **S1 Fig**.

### Mouse genotyping

DNA for PCR screening was extracted from adult ear and/or tail samples and from embryonic yolk sacs. Screening of Cre and Pitx2 floxed alleles was routinely done using used specific primers as previously described [16]. Cycling conditions for Cre were as follows; 5 min at 95°C, 35 cycles of 30s at 95°C, 30s at 60°C and 90s at 72°C, and for Pitx2 as follows; 5 min at 95°C. 40 cycles of 30s at 95°C, 30s at 60°C and 90s at 72°C, followed by a final extension step of 10 min at 72°C, respectively.

### Tissue and RNA isolation

When the experimental period was completed, rats were anaesthetized with thiobutabarbital (100 mg/kg IP, Inactin, Research Biochemicals International) and maintained at 37°C on a servo-controlled heated rodent operating table. Heart tissue samples corresponding to left (LA) and right (RA) atrial appendages and ventricular chambers were collected, processed accordingly to RNA isolation and stored at -80°C until used.

Genetically modified Pitx2 mice, and their corresponding controls, were sacrificed by cervical dislocation. Adult hearts were carefully dissected and briefly rinsed in Ringer’s solution. Tissue samples corresponding to the RA and LA were collected for each experimental condition, immediately snap-frozen in liquid nitrogen, and stored at -80°C until used. Pooled samples of at least three independent mice were processed for each condition, respectively. Three independent pooled samples were further processed for RNA isolation and qPCR analyses. Total RNA was isolated using Trizol (Roche) according to manufacture’s guidelines and DNase treated using RNase-Free DNase (Roche) for 1h at 30°C. In all cases, at least three distinct pooled samples were used to perform the corresponding qRT-PCR experiments.

First strand cDNA was synthesized at 50°C for 1h using 1 μg of RNA, oligo-dT primers and Superscript III Reverse Transcriptase (Invitrogen) according to manufacture’s guidelines. Negative controls to assess genomic contamination were performed for each sample, without reverse transcriptase, which resulted in all cases in no detectable amplification product.

### qRT-PCR (mRNA)

RT-PCR was performed in Mx3005Tm QPCR System with an MxPro QPCR Software 3.00 (Stratagene) and SyBR Green detection system. Reactions were performed in 96-well plates with optical sealing tape (Cultek) in 20 μL total volume containing SYBR Green Mix (Finnzymes) and the corresponding cDNA. Three internal controls, mouse βactin, Gusb and GAPDH, were used in parallel for each run and represented as previously described [14,33]. Amplification conditions were as follows: denaturisation step of 95°C for 10 min, followed by 40 cycles of 95°C for 30s, 60°C for 30s, 72°C for 30s; with final elongation step of 72°C for 10 min. All primers were designed to span exon-exon boundaries using online Primer3 software Primer3input (primer3 www.Cgi v 0.2). Primer sequences are provided in **S1 Table**. No amplifications were observed in PCR control reactions containing only water as the template. Each PCR reaction was performed at least three times to obtain representative averages. The Livak method was used to analyze the relative quantification RT-PCR data [33] and normalized in all cases taking as 100% the wild-type (control) value, as previously described [14].

### qRT-PCR (microRNA)

microRNA qRT-PCR was performed using Exiqon LNA microRNA qRT-PCR primers and detection kit according to manufacturer’s guidelines. All reactions were always run in triplicates using 5S as normalizing control, as recommended by the manufacturer. SyBR Green was used as quantification system on a Stratagene Q-Max 2005P qRT-PCR thermocycler. Relative measurements were calculated as described by Livak & Schmittgen [33] and control measurements were normalized to represent 100% as previously described [14].

### Plasmid, microRNA and siRNA cell transfections

HL-1 cells (6 × 10^5^ cells per well) were transfected with plasmids containing expression constructs for Pitx2, Wnt8a (Addgene), Wnt11a (Addgene, Cambridge, MA, USA), premiR-29a, pre-miR-200 (Exiqon) or siRNA-Pitx2c, siRNA-Zfhx3, siRNA-Enpep, siRNA-Sod2 (Sigma, Aldrich, Munich, Germany) as previously described [14,34]. Primary cultures of mouse fetal (E17.5) cardiomyocytes were isolated using standard procedures [35], cultures accordingly and treated with T4 hormone as previously reported [36]. siRNA sequences are provided in **S1 Table**.

### Statistical analyses

For statistical analyses of datasets, unpaired Student’s t-tests were used. Significance levels or P values are stated in each corresponding figure legend. P < 0.05 was considered statistically significant.

## Results

### Pitx2c>Wnt>microRNA signaling is severely impaired in experimental hyperthyroidism (HTD) rat model

**Fig 1:**
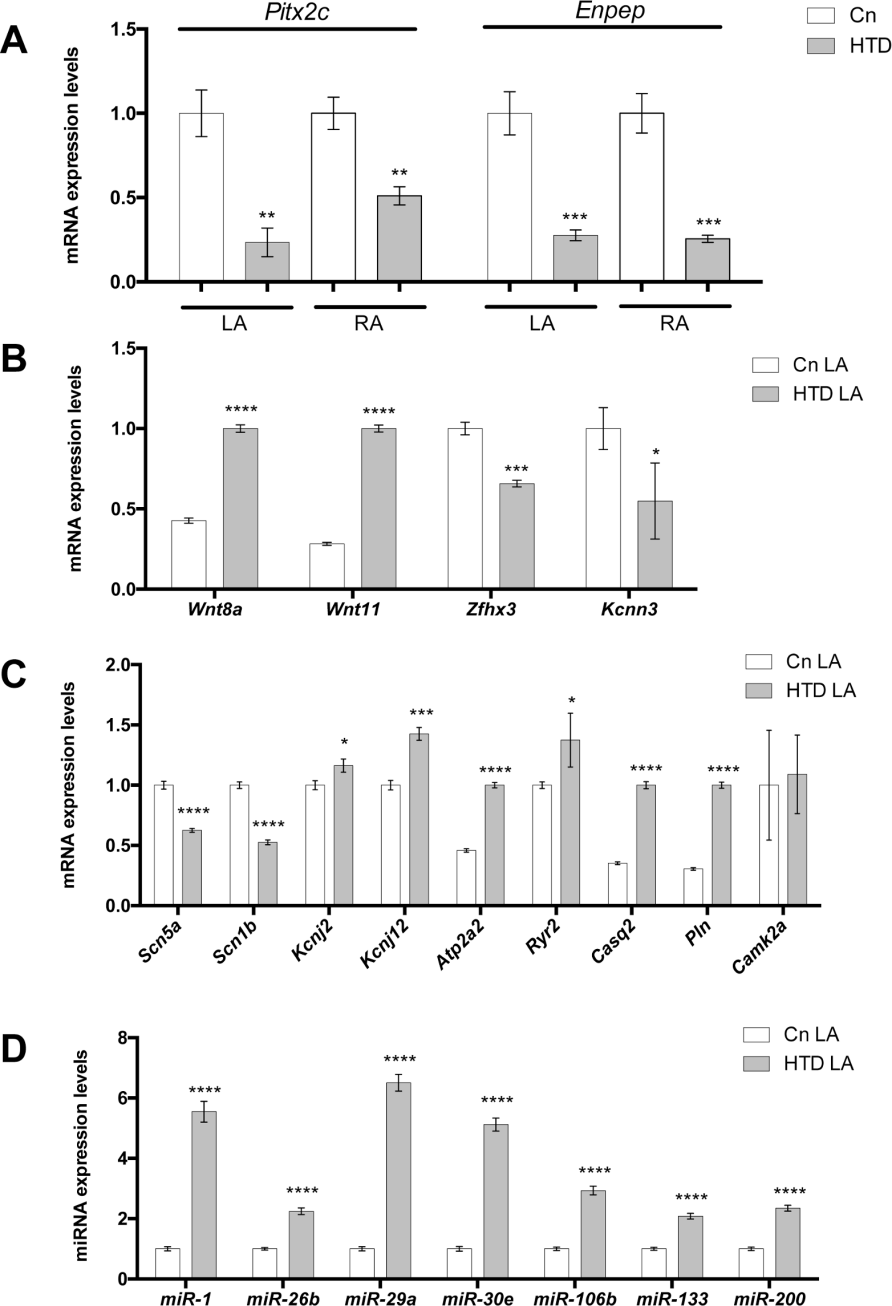
(A) *Pitx2* and *Enpep* gene expression in the left (LA) and right (RA) atrial chambers of experimental hyperthyroid rats (HTD) as compared to controls. Observe that *Pitx2* and *Enpep* are significantly decreased in both atrial chambers in HTD hearts. (B) AF GWAS-associated gene expression in the LA chamber of HTD hearts. Wnt8 and Wnt11 are significantly increased whereas Zfhx3 and Kcnn3 are decreased in HTD hearts as compared to controls. (C) Ion channel encoding genes display significant differential expression in HTD as compared to controls, while *Camk2a* display no significant differences. (D) Pitx2-regulated microRNAs display significant increased expression in HTD as compared to controls. In all cases, n = 6.*p<0.01, **p<0.05, ***p<0.001,****p<0.0001.

We have analyzed the expression levels of the homeobox transcription factor *Pitx2c* in an experimental rat model of induced hyperthyroidism (HTD) [32]. As a consequence of HTD, this experimental model also displays moderate hypertension as documented in **S1 Fig**. qPCR analyzed demonstrated that *Pitx2c* is severely down-regulated in right and left atrial chambers (**Fig 1**). Similar findings are also observed in an *in vitro* model of HTD (T4 administration) using fetal primary cultures of cardiomyocytes (**S2 Fig**). Curiously, *Enpep* is also severely impaired in both atrial chambers (**Fig 1**). Since multiple evidences have demonstrated that Pitx2 differentially modulates expression of AF GWAS associated genes as well as multiple components of the cardiac action potential in the left atrium [14,16], we analyzed their expression in the HTD model. Our data demonstrate that *Wnt8* and *Wnt11* were significantly increased whereas *Zfhx3* and *Kcnn3* were decreased in HTD rats as compared to age-matched controls (**Fig 1**). Importantly, I_Na_ encoding genes, i.e. *Scn5a* and *Scn1b*, are severely decreased whereas resting membrane potential I_K1_ encoding genes (*Kcnj2* and *Kcnj12*) are up-regulated, as well as calcium handling proteins such as *Atp2a2* (Serca), *Ryr2*, *Casq2* and *Pln* while *Camk2a* display no significant differences (**Fig 1**). Overall these data suggest that HTD elicits down-regulation of Pitx2 and its downstream signaling pathway, mimicking the gene expression profile observed in atrial-specific Pitx2 deficient mice [14,16].

A pivotal role for post-transcriptional regulation by non-coding RNAs in cardiac development and disease is emerging [37] and we have provided evidence that Pitx2 controls a large set of microRNAs with key functional roles in cardiac electrophysiology [15]. We therefore tested whether distinct Pitx2-regulated microRNAs were deregulated in our HTD rat model. qPCR of left atrial chambers demonstrated that miR-1, miR-26b, miR-29a, miR-30e, miR-106b, miR-133 and miR-200 are up-regulated in HTD rats as compared to controls (**Fig 1**), demonstrating a similar microRNA expression profile as in atrial-specific Pitx2 deficient mice [14,16].

### Pitx2 alone is impaired in experimental spontaneous hypertension (HTN)

**Fig 2:**
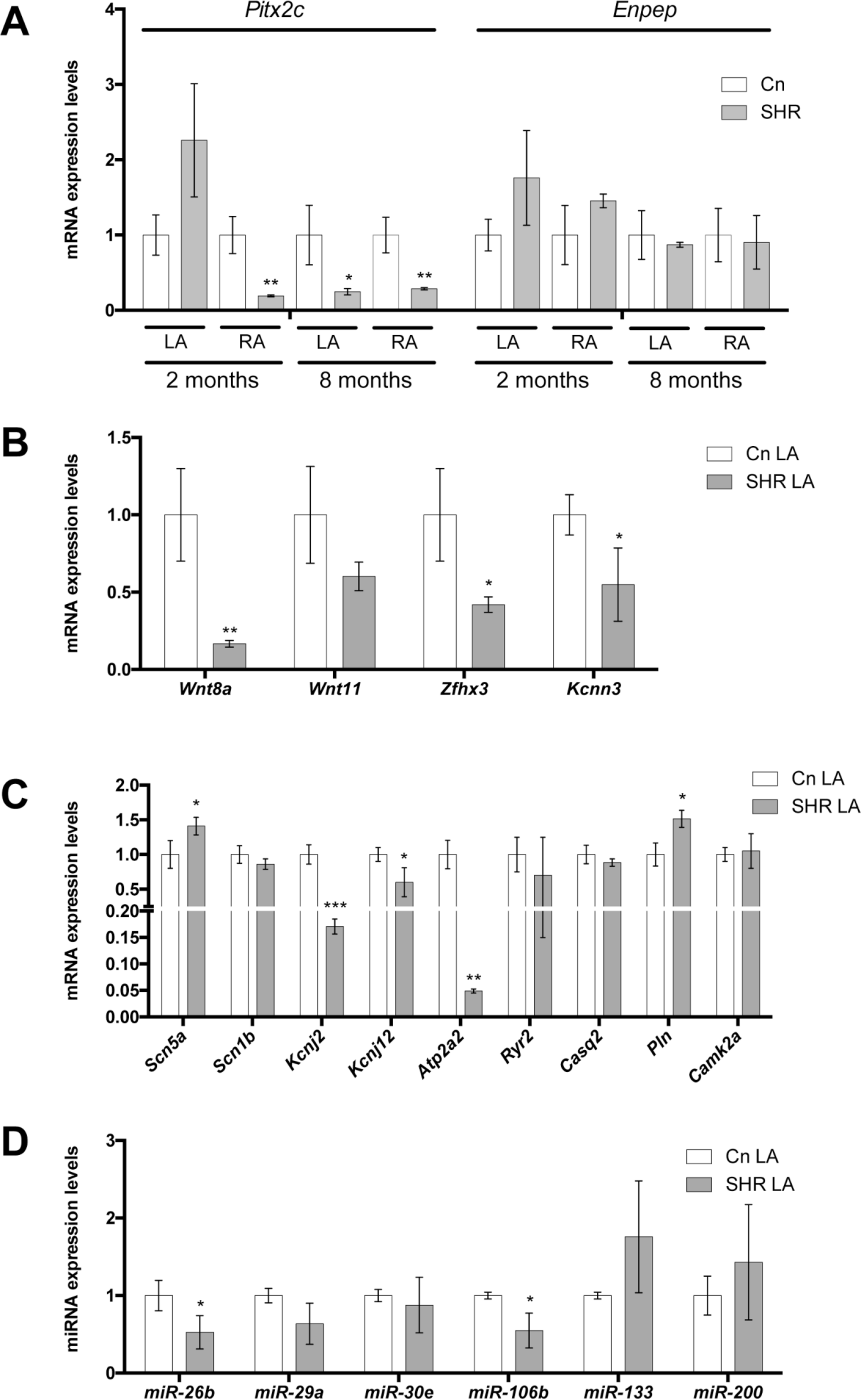
(A) *Pitx2* and *Enpep* gene expression in the adult left (LA) and right (RA) atrial chambers of spontaneous hypertensive rats (SHR) at 2 and 8 months, respectively, as compared to WHK controls. Observe that *Pitx2* is significantly decreased in both atrial chambers in SHR hearts at 8 months, whereas *Enpep* display no significant differences at any of the analyzed stages. (B) AF GWAS-associated gene expression in the LA chamber of HTD hearts. *Wnt8*, *Wnt11*, *Zfhx3* and *Kcnn3* are significantly decreased in HTD hearts as compared to controls. (C) Ion channel encoding genes such as *Kcnj2*, *Kcnj12*, *Atp2a2* (decreased), *Scn5a* and *Pln* (increased) display significant differential expression in HTD as compared to controls, while *Scn1b*, *Ryr2*, *Casq2* and Camk2a display no significant differences. (D) Pitx2-regulated microRNAs display no significant differences, except for miR-26b and miR-106b which are significantly decreased, in HTD as compared to controls. In all cases, n = 6. *p<0.01, ** p<0.05, *** p<0.001.

We further explored if *Pitx2c* is also altered in another AF risk factor experimental model, i.e. spontaneous hypertensive SHR rats [38]. We therefore analyzed *Pitx2c* expression levels at two distinct developmental stages, 2 and 8 month old rats, respectively. Interestingly, *Pitx2c* expression was significantly decreased in right atrium but not in the left atrium of 2 month old SHR rats, whereas down-regulation was equally observed at 8 months (**Fig 2**). Curiously, *Enpep* expression was not significantly different in left and right atrial chambers at any of these stages (**Fig 2**). Since most significant differences on *Pitx2* expression were only observed at 8 months, subsequent analyses were only performed at this stage.

Analyses of AF GWAS associated genes demonstrate that *Wnt8*, *Zfhx3* and *Kcnn3* are significantly decreased in hypertensive rats whereas *Wnt11* display no significant difference in the left atrial chambers of HTN rats as compared to controls (**Fig 2**). In addition, analyses of ion channel encoding genes demonstrate that *Scn5a*, but not *Scn1b* was significantly increased (**Fig 2**). *Kcnj2*, *Kcnj12* and *Atp2a2* (Serca2) are down-regulated whereas *Ryr2*, *Casq2* and *Camk2a* display no significant differences (**Fig 2**) and only *Pln* is up-regulated in HTN left atrial chambers as compared to control normotensive rats (**Fig 2**). Thus, although Pitx2 is severely down-regulated in HTN rats, Pitx2-downstream signals, such as Wnt signaling or cardiac action potential determinants display either no changes or discordant changes as compared to atrial-specific Pitx2 deficient mice [14,16].

Following the same reasoning as for HTD experimental model, we assessed if Pitx2-downstream microRNA expression is impaired in HTN rats. Surprisingly none of the tested microRNAs, except for *miR-26b* and *miR-106b*, which in fact were decreased, display significant differences (**Fig 2**). Overall, these data demonstrate that while Pitx2 is impaired in HTN, its downstream pathways are mostly unaltered, demonstrating a discordant microRNA expression profile as compared to those revealed in atrial-specific Pitx2 deficient mice [14,16].

### Enpep downregulation modulates PITX2 but not its downstream pathway

**Fig 3:**
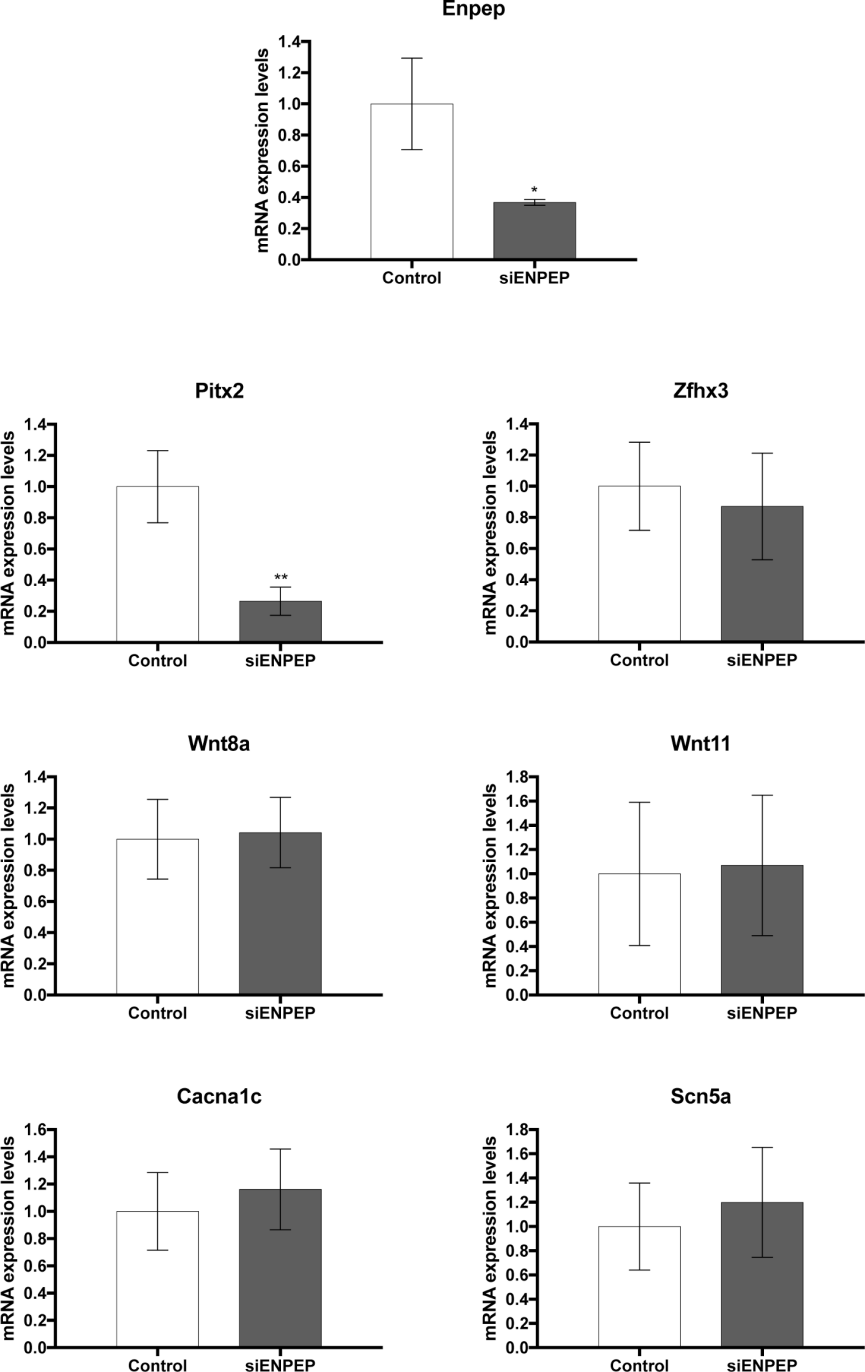
Gene expression analyses in Enpep silenced HL-1 atrial cardiomyocytes. Note that Enpep is decreased (siEnpep), leading to decreased expression of *Pitx2c* but not of *Wnt8a*, *Wnt11*, *Zfhx3*, *Cacna1c* or *Scn5a*. Representative values of three pooled replicates on three independent biological transfections. *p<0.01, **p<0.05.

Given the fact that Enpep is deregulated in HTD experimental rats, it is widely expressed in cardiac regions that can contribute to AF and that genomic interaction between 4q25 AF risk variants containing sequences and the Enpep promoter have been reported in mice [11], it is plausible that ENPEP might have a role in AF predisposing factors. Furthermore, Pitx2 silencing in HL1 atrial cardiomyocytes also elicits *Enpep* downregulation (**S2 Fig**). We therefore silenced *Enpep* expression in HL1 atrial cardiomyocyte and evaluated expression of *Pitx2c* and Pitx2-Wnt downstream signaling. Our analyses demonstrated that *Enpep* silencing decreased *Pitx2* expression whereas *Zfhx3*, *Wnt8*, *Wnt11*, *Cacna1c* and *Scn5a* were unaltered (**Fig 3**). Thus these data demonstrate that Enpep contribution on the Pitx2-Wnt signaling pathways is limited to Pitx2 regulation.

### Impaired ROS signaling contributes to Pitx2 pathophysiology

**Fig 4:**
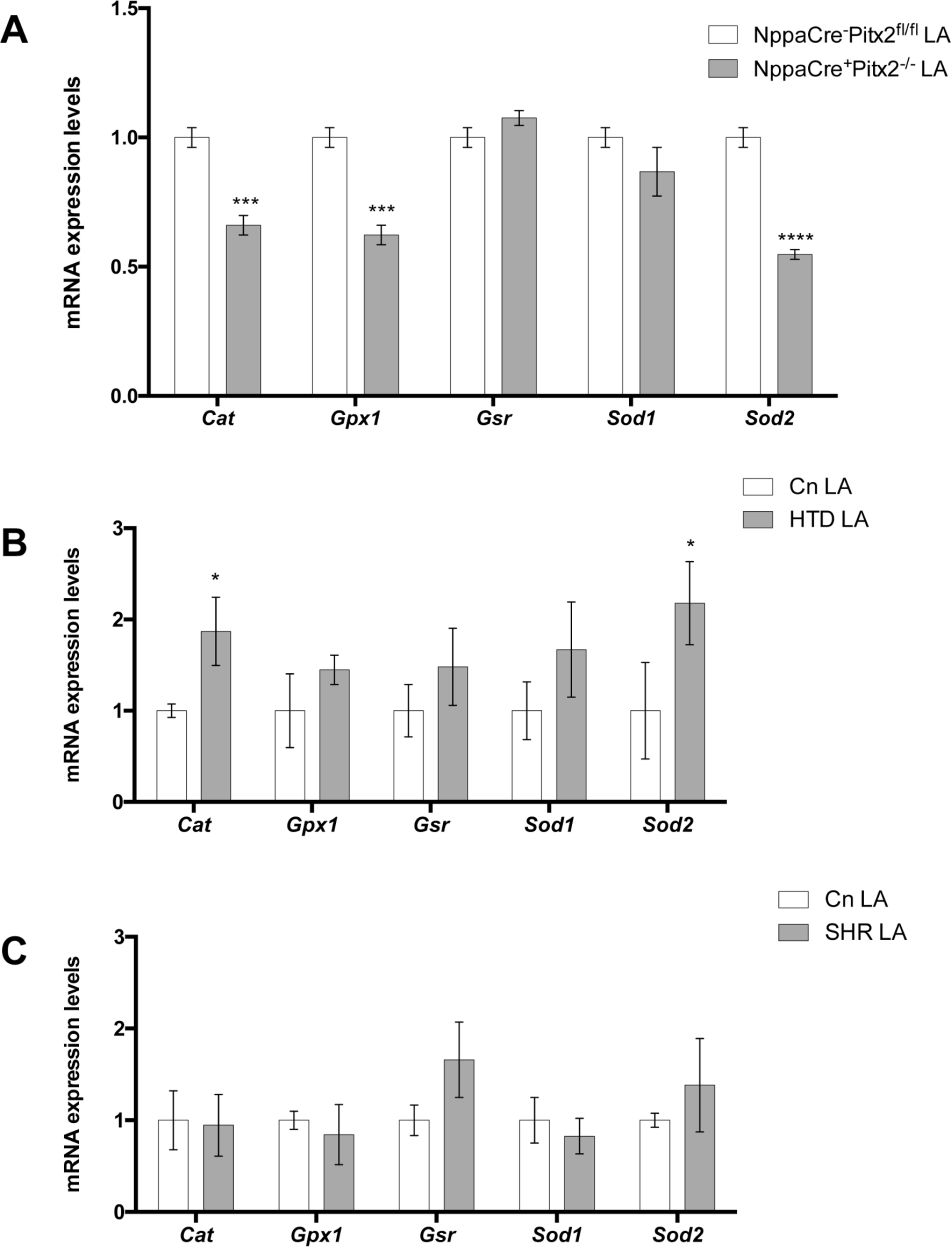
Redox signaling gene expression analyses of adult left atria (LA) corresponding to NppaCre^+^Pitx2^-/-^ (A), HTD (B) and SHR (C), respectively, as compared to their corresponding controls. Note that *Cat*, *Gpx1* and *Sod2* are significantly decreased in NppaCre^+^Pitx2^-/-^ as compared to NppaCre^-^Pitx2^fl/fl^ controls, *Cat* and *Sod2* are significantly increased in HTD mice whereas no changes are observed in SHR mice. In all cases, n = 6. *p<0.01, ***p<0.001, ****p<0.0001.

Reactive oxidative signaling (ROS) has been recently linked to AF [20,39] and experimental evidences demonstrated that impairing ROS can act as pro-arrhythmogenic factor [40]. We have therefore investigated if ROS is impaired in our atrial-specific Pitx2 mouse mutants as well as in AF risk factor HTD and SHR experimental models. qPCR demonstrate that several components of ROS signaling are impaired. In particular, we noticed that catalase (*Cat*), glutathione peroxidase (*Gpx)* and mitochondrial superoxide dysmutase (*Sod2*) were significantly down-regulated in the left atrial chamber of atrial-specific Pitx2 mouse mutants as compared to controls, whereas cytoplasmic superoxide dysmutase (*Sod1*) and glutathione reductase (*Gsr*) were unaltered (**Fig 4**). Subsequently we tested if ROS is impaired in SHR and/or HTD experimental models. Interestingly, HTD leads to up-regulation of *Cat* and *Sod2* but not any of the other ROS analyzed components (**Fig 4**) whereas HTN (SHR) elicited no significant changes in any of the studied ROS components (**Fig 4**). These data illustrate that Pitx2 insufficiency leads to altered ROS signaling, a condition that is minimally (HTN) or partially (HTD) impaired, respectively.

**Fig 5:**
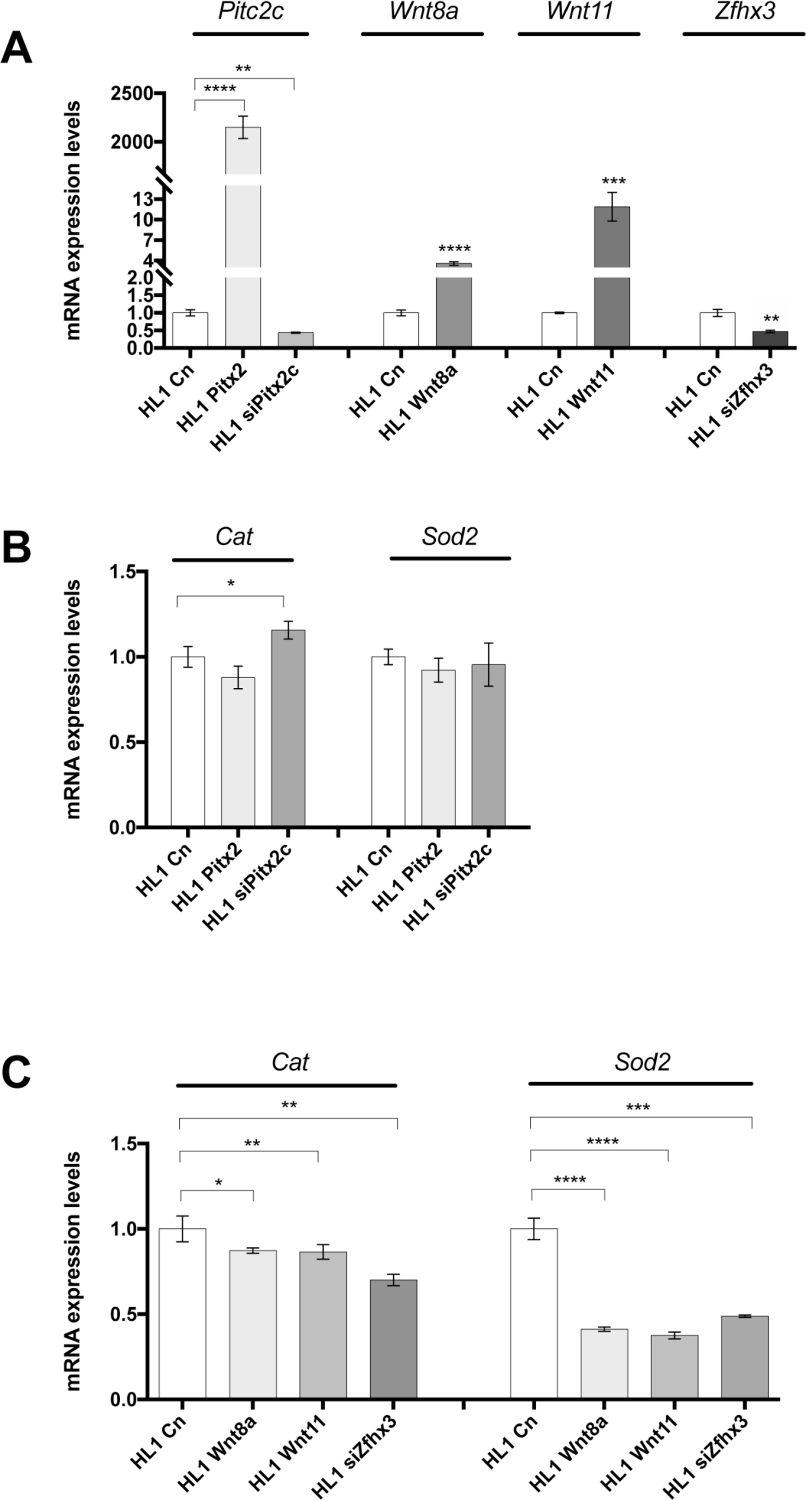
(A) Expression analyses of Pitx2c, Wnt8a, Wntt11 and Zfhx3 in gain- and loss-of-function experimental transfection of HL-1 atrial cardiomyocytes for Pitx2, Wnt8a, Wnt11 and Zfhx3, respectively. (B) Expression analyses of *Cat* and *Sod2* in *Pitx2* gain and loss-of-function experimental transfection of HL-1 atrial cardiomyocytes. Observe that only Pitx2c silencing (siPitx2c) significantly increases Cat expression. (C) Expression analyses of *Cat* and *Sod2* in Wnt8a and Wnt11 gain of function transfections and Zfhx3 siRNA silencing (siZfhx3) of HL-1 atrial cardiomyocytes. Observe that *Cat* and *Sod2* are significantly decreased in all cases, as compared to controls. Representative values of three pooled replicates on three independent biological transfections. *p<0.01, **p<0.05, ***p<0.001, ****p<0.0001.

To test if *Pitx2c* can directly modulate ROS components, Pitx2 over-expression and silencing (siRNA) experiments were carried out in HL-1 atrial cardiomyocytes (**Fig 5**). Unexpectedly, Pitx2 over-expression did not influence *Cat* and *Sod2* expression whereas Pitx2 silencing slightly up-regulated *Cat* but not *Sod2*, as depicted in **Fig 5**. These data are in contrast with our previous findings in atrial-specific Pitx2 mutant mice (**Fig 4**). Thus it could be possible that Pitx2 does not directly target *Cat*/*Sod2* expression but else is modulated downstream of Pitx2. Mimicking Pitx2 insufficient model, we thus over-expressed Wnt8 and Wnt11, respectively and inhibited Zfhx3 by using siRNA in HL-1 atrial cardiomyocytes. Interestingly, both over-expression of Wnt signaling (Wnt8 and Wnt11) and inhibition of Zfhx3 (**Fig 5**), respectively, lead to significant downregulation of *Cat* and *Sod2* expression (**Fig 5**). These data therefore demonstrate that ROS signaling is modulated by Wnt and Zfhx3 signaling downstream Pitx2.

**Fig 6:**
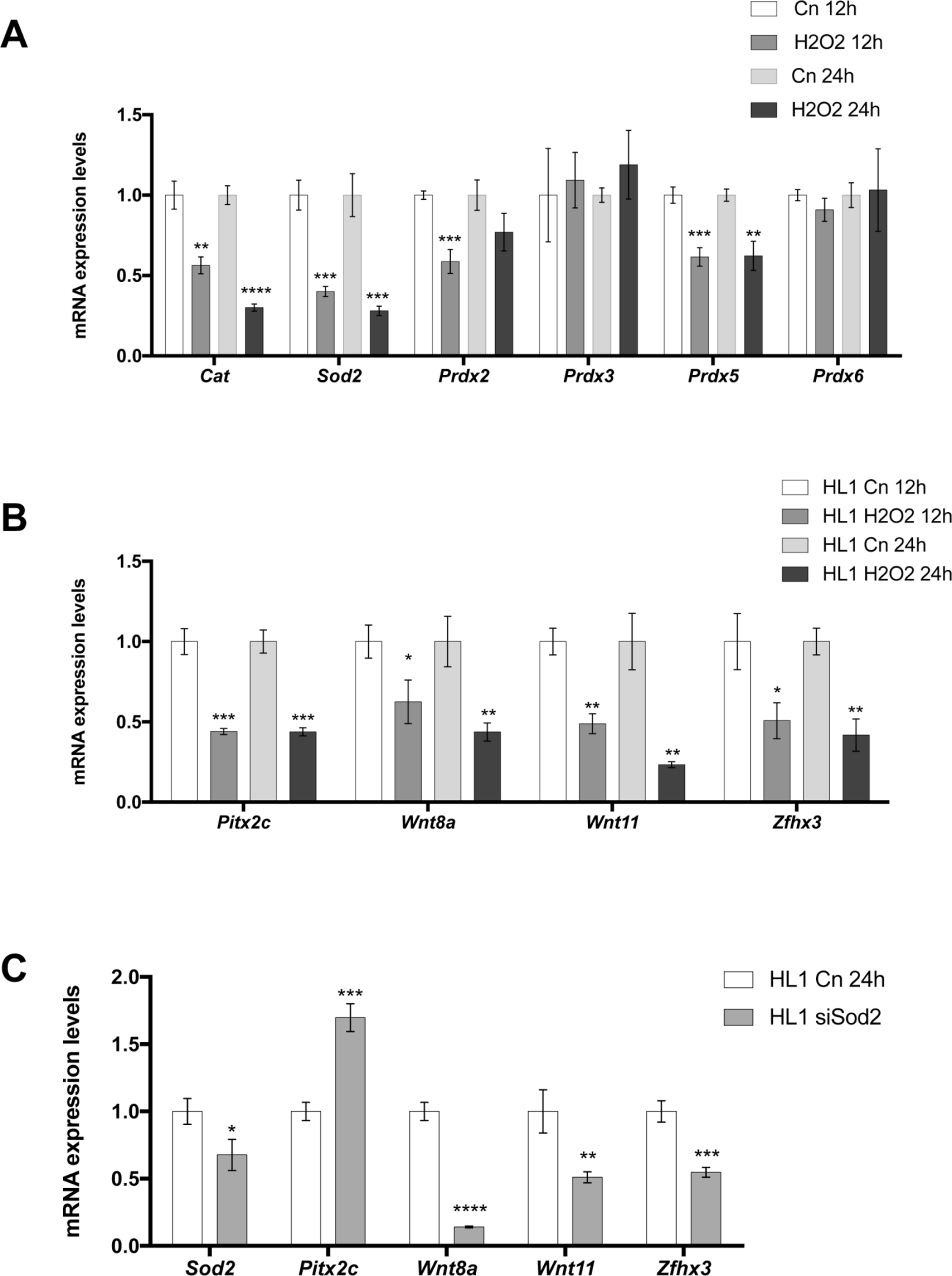
(A) Redox signaling gene expression analyses of H_2_0_2_ treated HL-1 atrial cardiomyocytes at 12 h and 24h. Observe that *Cat*, *Sod2* and *Prdx5* are significantly decreased at 12h and 24h after H_2_0_2_ administration, whereas *Prdx2* is only decreased at 12h but not at 24h. (B) Gene expression analyses of *Pitx2c*, *Wnt8a*, *Wnt11* and *Zfhx3* after H_2_0_2_ administration. Observe that all of them are significantly decreased at 12h and 24h. (C) Gene expression analyses in Sod2 silenced HL-1 atrial cardiomyocytes. Note that Sod2 is decreased (siSod2), *Pitx2c* increases whereas *Wnt8a*, *Wnt11* and *Zfhx3* are significantly decreased. Representative values of three pooled replicates on three independent biological transfections. *p<0.01, **p<0.05, ***p<0.001, ****p<0.0001.

To test if the opposite pathways is operative, i.e. if ROS signaling alters Pitx2>Wnt pathway, we treated HL-1 atrial cardiomyocytes with hydrogen peroxidase (H_2_O_2_) at distinct incubation times ranging from 1h to 24h and we assessed if Pitx2, Wnt signaling and Zfhx3 is impaired. *Cat*, *Sod2* and also peroxiredoxines (*Prxd2*, *Prdx3*, *Prdx5* and *Prdx6*) were assayed in parallel as indicators of ROS signaling activity. Incubation times ranging from 1h to 6h display basically no significant changes in the expression level (data not shown), except for a transient up-regulation of *Wnt11* (3h/6h; **S3 Fig**). Curiously, sustained down-regulation of *Zfhx3* expression was observed at all time analyzed (**S3 Fig**). At 12h/24h of H_2_0_2_ administration *Cat*, *Sod2*, *Pdrx2* (only 12h) and *Prdx5* were significantly decreased in treated cells as compared to controls, whereas *Prdx3* and *Prdx6* display no significant differences (**Fig 6**). These data suggest therefore that impaired ROS signaling was successfully achieved at 12h/24h of H_2_0_2_ administration. Importantly, *Pitx2*, *Wnt8*, *Wnt11* and *Zfhx3* were severely down-regulated after H_2_0_2_ administration at both 12h/24h (**Fig 6**). Thus these data demonstrate that ROS impairment can influence Pitx2>Wnt signaling. To further support these findings we selectively inhibited Sod2 expression by siRNA in HL1 atrial cardiomyocyte and assayed Pitx2>Wnt signaling components by qPCR. Sod2 inhibition leads to down-regulation of *Wnt8a*, *Wnt11* and *Zfhx3* and surprisingly to up-regulation of *Pitx2* as depicted in **Fig 6**. While up-regulation of *Pitx2c* after Sod2 siRNA remains to be fully understood, our data reveal a complex interplay between Pitx2>Wnt and ROS signaling in atrial cardiomyocytes.

**Fig 7:**
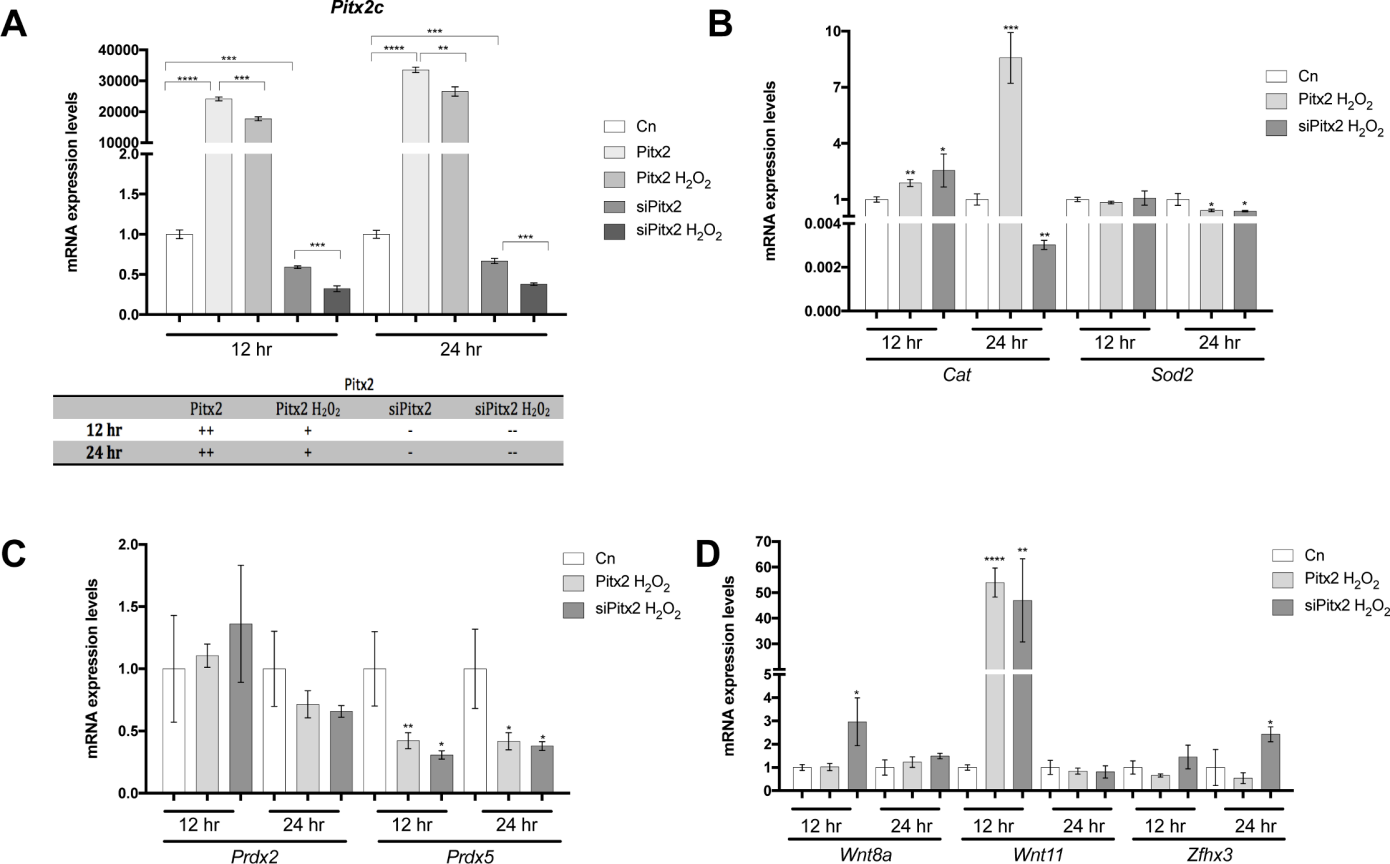
(A) Analyses of *Pitx2c* expression in Pitx2 gain and loss-of-function experiments with or without H_2_0_2_ administration for 12h and 24h, respectively. Observe that H_2_0_2_ administration significantly decreased Pitx2c expression in both Pitx2 overexpression and silencing conditions, at 12h and 24h. (B) Analyses of *Cat* and *Sod2* expression (B), *Prdx2* and *Prdx5* (C) and *Wnt8a*, *Wnt11* and *Zfhx3* (D) in Pitx2 gain and loss-of-function setting with H_2_0_2_ administration for 12h and 24h, respectively. Observe that *Cat* is significantly increased by Pitx2 overexpression at 12h and 24h whereas is mildly increased and highly significantly decreased by Pitx2 siRNA treatment at 24h. On the other hand Sod2 is mildly decreased only at 24h in both conditions (B). *Prdx5*, but not *Prdx2*, is significantly decreased at 12h and 24h in both conditions (C). *Wnt8a* is only increased in Pitx2c siRNA conditions at 12h, *Wnt11* is increased in both conditions only at 12h while *Zfhx3* is significantly increased only by Pitx2c silencing at 24h. Representative values of three pooled replicates on three independent biological transfections. *p<0.01, **p<0.05, ***p<0.001, ****p<0.0001.

To further decipher the complex interplay between ROS and Pitx2 signaling, we experimentally test if Pitx2c gain- and loss-of-function was impaired by H_2_0_2_ administration in HL-1 atrial cardiomyocytes, aiming to decipher which is the overruling signaling pathway. Pitx2c transfection lead to over-expression of *Pitx2c*, which was significantly diminished by H_2_0_2_ administration at both experimental time points (12h/24h) (**Fig 7**). Similarly, *Pitx2c* expression was further inhibited by H_2_0_2_ administration after Pitx2c siRNA silencing experiments (**Fig 7**), further demonstrating that H_2_0_2_ administration significantly decreases *Pitx2c* expression.

In this experimental context, H_2_0_2_ administration does not alter *Sod2* expression in Pitx2c gain and loss-of-function settings at 12 hours but it significantly decreases it at 24 hours (**Fig 7**). On the contrary, *Cat* expression is enhanced by H_2_0_2_ administration in Pitx2 gain and loss-of-function settings at 12h but selectively decreased at 24hours only in Pitx2 loss-of-function conditions. These data suggest that H_2_0_2_ administration cannot overrule Pitx2 modulation of *Cat*. In line with these findings only *Prdx5* is significantly decreased after H_2_0_2_ administration in Pitx2c gain and loss-of-function settings at both 12 hours and 24 hours (**Fig 7; S4 Fig**), further supporting that H_2_0_2_ administration overrules Pitx2c modulation of ROS signaling components.

**Fig 8:**
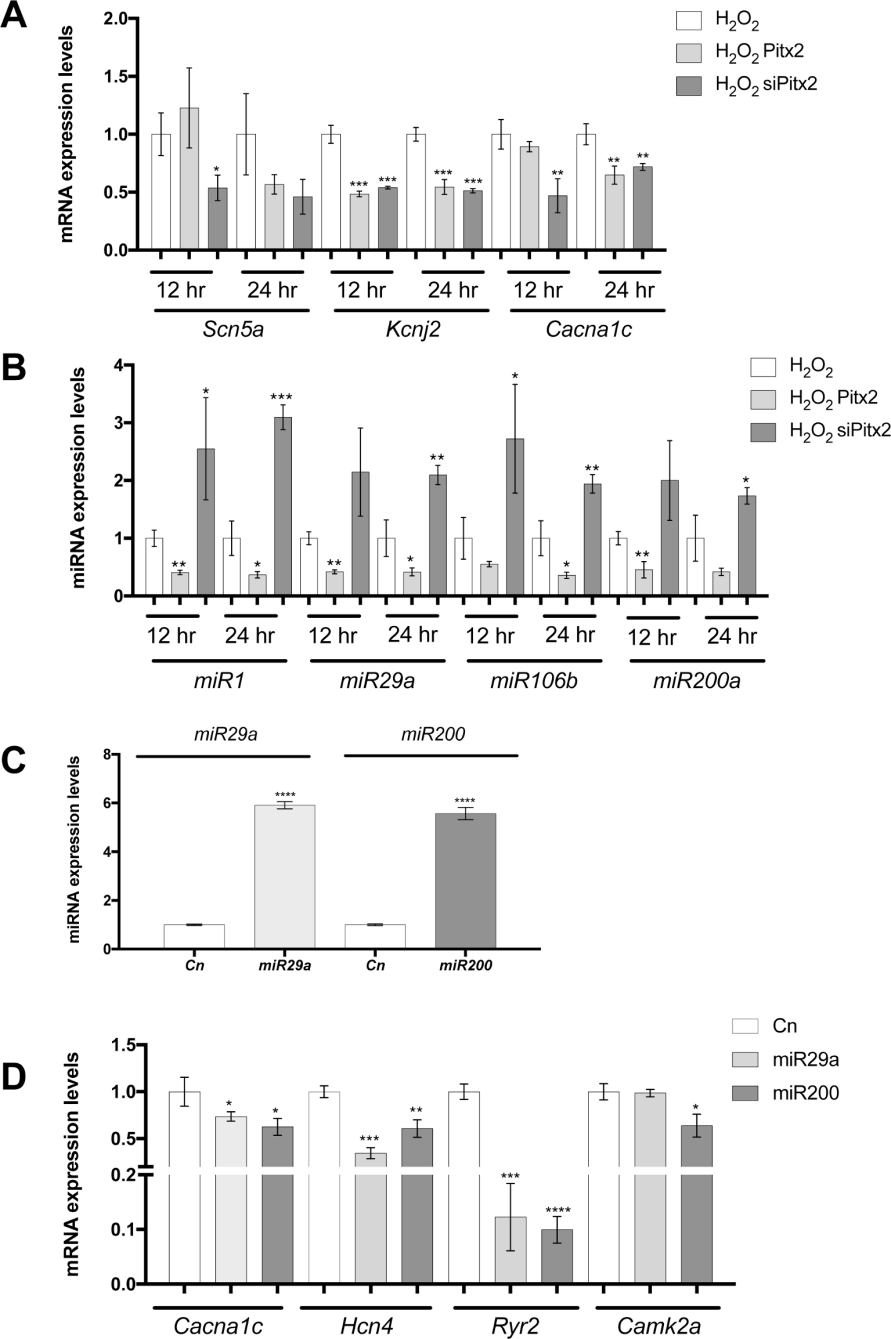
Analyses of *Scn5a*, *Kcnj2* and *Cacna1c* (A), *miR-1*, *miR-29a*, *miR-106b* and *miR-200b* (B) expression in Pitx2 gain and loss-of-function experiments after H_2_0_2_ administration for 12h and 24h, respectively. Observe that most ion channel are significantly decreased in both experimental conditions at both time points (A) whereas microRNAs are all significantly decreased after Pitx2 over-expression and increased following Pitx2c silencing at 12h and 24h after H_2_0_2_ administration. (C). *miR-29a* and *miR-200* expression in HL-1 atrial cardiomyocytes transfected cells. Observe that each microRNAs is significantly increased after corresponding pre-miRNA transfection. (D) Ion channel gene expression in *miR-29a* and *miR-200* transfected HL-1 atrial cardiomyocytes, respectively. Observe that miR-29 and miR-200 over-expression leads to significant decreased of *Cacna1c*, *Hcn4*, *Ryr2* and *Camk2a* (except for miR-29a) expression. Representative values of three pooled replicates on three independent biological transfections. *p<0.01, **p<0.05, ***p<0.001, ****p<0.0001.

In order to investigate if H_2_0_2_ administration can also overrule Pitx2 modulation of its downstream targets, *Wnt8a*, *Wnt11* and *Zfhx3* expression was analyzed in this experimental setting. As it can be observed in **Fig 7**, H_2_0_2_ administration prevented *Wnt8* and *Wnt11* up-regulation in Pitx2c siRNA silenced conditions at 24h but not at 12hours, demonstrating that H_2_0_2_ administration can also overruled Wnt signaling up-regulation in absence of Pitx2c. On the other hand, *Zfhx3* was up-regulated after H_2_0_2_ administration in Pitx2c silenced conditions at 24hours, demonstrating that H_2_0_2_ administration can directly affect *Zfhx3* expression independently of Pitx2c expression levels. Overall these data further demonstrate that ROS signaling has a major impact on Pitx2>>Wnt signaling pathway. We further analyzed if ion channel expression would be affected in this experimental setting. qPCR analyses of *Cacna1c*, *Scn5a* and *Kcnj2* expression demonstrated significant down-regulation at both 12 and 24 hours after H_2_0_2_ administration in Pitx2c silenced conditions (**Fig 8**). Importantly, such H_2_0_2_ mediated down-regulation was only observed for *Kcnj2* in Pitx2c over-expressing cells, demonstrating a fine output balanced between ROS and Pitx2 function on the expression of distinct ion channels. Finally, we tested if microRNA expression would be also impaired in this context. A subset of Pitx2-modulated microRNA was assessed^16^. As depicted in **Fig 8**, *miR-1*, *miR-29a*, *miR-106b* and *miR-200a* was selectively inhibited by H_2_0_2_ treated Pitx2-overexpressing cells but up-regulated in H_2_0_2_ treated Pitx2 silenced cells at both time points (12h and 24h). These data illustrate that H_2_0_2_ administration does not overrule Pitx2 function as regulator of microRNA expression.

### Modulation of miR-29 and miR-200 alters cardiac action potential determinants

We have previously demonstrated that Pitx2 modulates expression of *miR-29* and *miR-200*, among other microRNAs [16] and furthermore we have demonstrated in this study that modulation of distinct ion channel is greatly influenced by H_2_0_2_ administration while microRNA signature is mostly dependent on Pitx2c but not H_2_0_2_ administration. We provide herein evidences that *miR-29* and *miR-200* over-expression also contributes to ion channel expression remodeling. HL-1 atrial cardiomyocytes transfected with *miR-29* and *miR-200* (**Fig 8**) significantly down-regulate *Cacna1c*, *Hnc4* and *Ryr2* expression, while *Camk2a* was significantly decreased with *miR-200* but not *miR-29* (**Fig 8**). Thus these data demonstrate that *miR-29* and *miR-200* impaired expression also contributes to develop pro-arrhythmogenic substrates.

## Discussion

Multiple risk factors are associated with the onset of AF [23], however understanding of the molecular causative links remains poorly elucidated. Among these risk factors, hyperthyroidism is highly linked to increased prevalence of AF [41–43]. It is widely documented that electrical properties of the atrial cardiomyocytes are functional impaired in hyperthyroid patients [41] as well as in distinct experimental models of hyperthyroidism [44–47]. In particular, there are ample evidences that TH deregulation can influence calcium and potassium channels [44–47], whereas modulation of sodium channels is less documented [48]. Similarly, AF onset is also increased in HTN patients [23,49]. While extensive literature is available on the modulation of distinct ion channels in the vasculature of hypertensive patients [50–51], scarce evidence is available on cardiac ion channel deregulation and thus electrical impairment in the heart. Recently, generation of reactive oxygen species (ROS) has been associated to increase onset of AF [52–53] and dietary ROS reduction has been provided to be beneficial [54] on AF onset. Substantial evidence support ROS regulation of distinct cardiac ion channels [55–57], procuring thus a functional link between ROS signaling and impaired electrical activity leading to AF onset.

Thus, whereas there are evidence of functional impairment by these cardiovascular risk factors leading to AF, it largely remains unclear which are the molecular mechanisms driving AF in these contexts. Multiple point mutations in distinct ion channels have been reported to contribute to AF pathophysiology [58] yet covering less than 10% of AF cases. Genome-wide association studies have brought up novel candidate genes on AF pathophysiology among which most significantly associated risk variants are in the vicinity of the homeobox transcription factor PITX2 [6]. Genomic loci spanning within risk variants are capable of molecularly interacting with PITX2 as well as ENPEP regulatory elements [11], yet the specific role of these risk variants in AF remains elusive. Importantly, distinct Pitx2 loss-of-function experimental models demonstrated that this transcription factor controls a complex molecular signaling pathway that substantially modulates expression of multiple genes encoding ion channel and cell-cell proteins with pivotal role in cardiac electrophysiology [12–26]. We provide here first evidence that hyperthyroidism leads to decreased expression of PITX2 and ENPEP in the atrial chambers. Furthermore, we also demonstrated for the first time that impaired ROS signaling modulates PITX2 expression, while we corroborate previous findings that PITX2 expression, but not ENPEP, is significantly down-regulated in hypertensive rats [28]. Therefore these data demonstrate that PITX2 expression is impaired in HTD, a cardiovascular risk factor leading to AF, suggesting that PITX2 impairment could be pivotal provoking atrial arrhythmogenesis.

Pitx2 insufficiency in mice has been reported to distinctly modulate Sox2-Hcn4 expression [12] in the developing embryo leading to impaired conductive configuration and thus predisposition to AF. In addition, Pitx2 insufficiency in the adult heart has been proven to regulate I_Na_ and I_K1_ currents, leading to impaired ECG [14] and additionally to regulate calcium homeostasis by modulating Wnt signaling in a dose-dependent manner [16]. We provide herein evidences that HTD increases Wnt8/Wnt11 expression in the left atrial chamber while down-regulates Zfhx3 expression, mimicking thus similar results obtained in Pitx2 insufficient mice. Furthermore, genes encoding I_Na_ (*Scn5a*, *Scn1b*) were down-regulated, those encoding for I_K1_ (*Kcnj2*, *Kcnj12*) were up-regulated as well as those controlling calcium handling (*Ryr2*, *Atp2a2*, *Casq2*, *Plb*) were also up-regulated, in line with previous findings in atrial-specific conditional Pitx2 insufficient mice. Interestingly, such up-regulated Wnt signaling was not observed in the left atrial chamber of SHR rats (HTN model), neither such ion channel impaired expression. While Enpep is differentially expressed in HTN vs SHF left atrial chambers, Enpep provides no contribution to Wnt signaling or downstream ion channel expression. A plausible explanation might be that hypertension directly influences Wnt expression [59], counteracting Pitx2-mediated Wnt up-regulation. Importantly, HTD rats also course with mild hypertension, supporting the notion that the specific molecular changes observed in HTD but not in SHR (HTN) are modulated by thyroid hormones. Additional experiments will be needed to further dissect these pathways.

We and other have previously reported the pivotal role of microRNAs as post-transcriptional regulatory mechanism driven by Pitx2 in the context of atrial arrhythmogenesis [57–58], and we therefore analyzed if expression of distinct cardiac enriched microRNAs were impaired in HTD and SHR (HTN model) rats. Importantly, we have observed that microRNA profiling in the left atrium of HTD, but not in SHR, rats, mimicking previous findings in atrial-specific conditional Pitx2 insufficient mice [16]. Several lines of evidence have already reported the key regulatory role of miR-1 [60–62], miR-26 [63], miR-106b [64], miR-133 [65–66] and miR-200 [64] in arrhythmogenesis. We provide herein additional evidence that these Pitx2-modulated HTD-regulated microRNAs modulate distinct ion channel expression with relevance in atrial electrophysiology. miR-29 over-expression in HL1 atrial cardiomyocyte deregulate *Cacna1c*, *Hnc4* and *Ryr2*, influencing therefore both the calcium handling and pacemaker activity, whereas miR-200 regulated *Cacna1c*, *Ryr2* and *Camk2a*, in addition to *Scn5a* as previously reported [64], impacting therefore also in calcium handling. Importantly, miR-29 and miR-200 are not significantly impaired in SHR atrial chambers, suggesting that Wnt-microRNA might be a pivotal candidate establishing fundamental differences between HTD and HTN in atrial arrhythmogenesis susceptibility.

It has been demonstrated that Pitx2 play a pivotal role regulating redox homeostasis in the adult skeletal muscle as well as during skeletal muscle regeneration [28]. More recently, Pitx2 has been demonstrated to play a pivotal role promoting resistance to ischemia by activating an antioxidant response in the adult heart [29]. We provide herein evidences of a complex interplay between Pitx2 and redox signaling. On the one hand, Pitx2 insufficiency leads to significant expression impairment of key redox components, such as *Cat* (catalase) and *Gpx* (Glutathione reductase) and *Sod2* (mitochondrial superoxide dismutase), in line with previous findings [28,29]. On the other hand, impaired redox homeostasis also significantly alters Pitx2 and its downstream AF signaling pathway, i.e. Wnt and Zfhx3 expression. Importantly, Wnt8/Wnt11 and Zfhx3 expression display significant decreased expression levels in both H_2_O_2_ and siRNA Sod2 atrial cardiomyocytes. Interestingly, redox signaling impairment is also observed in HTD but not in SHR (HTN model) rats, suggesting that redox impairment in the context of HTD can also contribute to atrial arrhythmogenesis in this setting.

Given the complex interplay between redox homeostasis and Pitx2 signaling, we have analyzed the molecular consequences of deregulating both pathways in atrial cardiomyocytes. We noticed that redox impairment significantly elicits down-regulation of Pitx2 independently of its expression levels, i.e. controls, Pitx2 over-expressing and Pitx2 silenced atrial cardiomyocytes. Furthermore, sustained H_2_O_2_ administration (24h) significantly blocked Pitx2-repressed Wnt expression and promotes Zfhx3 up-regulation. Whereas it is widely documented that redox signaling can compromise ion channel functioning and calcium homeostasis in cardiomyocytes [67], in our system we observed no influence of H_2_O_2_ administration on the regulatory impact of Pitx2 in distinct ion channels such as *Scn5a*, *Kcnj2* and *Cacna1c* as well as multiple Pitx2-regulated microRNAs such as miR-1, miR-26, miR-29 and miR-200, in which redox impairment impact is less documented [68].

**Fig 9:**
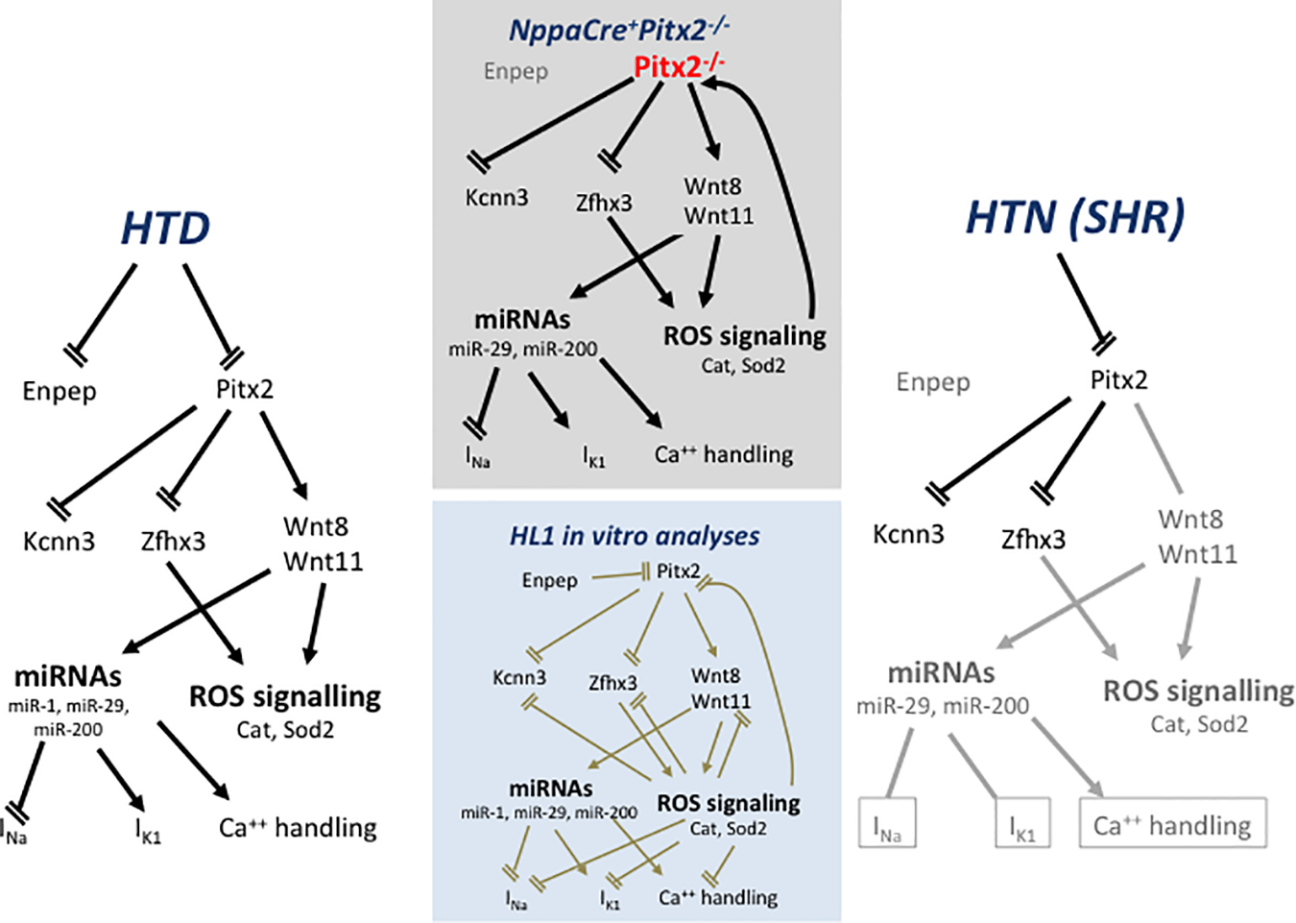
Schematic representation of the Pitx2>Wnt>ROS signaling pathway in NppaCrePitx2-/- insufficiency mice, as compared to experimental HTD and HTN (SHR) rat models. Observe that Pitx2 insufficiency leads deregulation of Zfhx3 and Wnt signaling, which subsequently leads to microRNA and ROS signaling deregulation and thus ion channel impairment. In HTD but not in HTN, Pitx2>Wnt>ROS signaling is also impaired. Furthermore, *in vitro* gain- and loss-of-function analyses (brown arrows) further support a complex retroactive gene regulatory network.

In summary, we provide herein evidences that Pitx2-Wnt signaling pathway is impaired in HTD rats (**Fig 9**). Comparative analyses with Pitx2 insufficient models highlights the molecular hallmark similarities between HTD and atrial-specific Pitx2 deficient hearts, in particular Wnt upregulation, Zfhx3 down-regulation and redox signaling impairment. These data therefore support the notion that AF onset in HTN patients is mediated by Pitx2 impairment. Furthermore, we provide evidences of a complex interplay between Pitx2 and redox signaling highlighting therefore the complex molecular interactions priming molecular substrates that contribute to atrial arrhythmogenesis.

## Supporting information

S1 Table List of oligonucleotides for mRNA and microRNA detection by qPCR as well as oligonucleotide sequences used for siRNA silencing of *Enpep*, *Sod2* and *Pitx2c*, respectively. (PDF)

S1 Fig A) Systolic blood pressure (SBP) in mmHg and heart rate (HR) in beats per minute (BPM) measured in control and hyperthyroid (HTD) rats at the end of the experiment (n = 10 each group). Mean ± SEM is displayed. *p<0.001 *vs* control group. B) Systolic blood pressure (SBP) in mmHg and heart rate (HR) in beats per minute (BPM) measured in Wistar-Kyoto (WKY) and spontaneously hypertensive rats (SHR) at the end of the experiment (n = 10 each group). Mean ± SEM is displayed. *p<0.01, **p<0.001 *vs* control group.
(TIFF)

S2 Fig Analyses of Pitx2 expression in primary culture of fetal cardiomyocytes treated with T4 as compared to controls (panel A). Observe that Pitx2 is significantly decreased after T4 administration. Analyses of Pitx2 (panel B) and Enpep (panel C) in HL1 atrial cardiomycytes after Pitx2 siRNA silencing. Observe that Pitx2 siRNA administration significantly decrease Pitx2 expression (panel B) and also Enpep expression (panel C). *p<0.01, ***p<0.001, ****p<0.0001.
(TIF)

S3 Fig Analyses of *Wnt11* and *Zfhx3* expression in H_2_0_2_ treated HL-1 atrial cardiomyocytes at 1h, 3h and 6h, respectively. Observe that H_2_0_2_ administration significantly increased *Wnt11* at 3h and 6h while significantly decreased *Zfhx3* expression at all experimental conditions analyzed. *p<0.01, **p<0.05, ****p<0.0001.
(TIF)

S4 Fig Analyses of *Prdx3* and *Prdx6* expression in Pitx2 gain and loss-of-function experiments with or without H_2_0_2_ administration for 12h and 24h, respectively. Observe that no significant differences are observed in *Prdx3* and *Prdx6* expression, except for *Prdx6* at 12h after treatment in Pitx2 silencing conditions. *p<0.01.
(TIF)
