# Supplementary material for: Hyperthyroidism, but not hypertension, impairs PITX2 expression leading to Wnt-microRNA-ion channel remodeling
Source: PLoS One. 2017 Dec 1;12(12):e0188473. doi: 10.1371/journal.pone.0188473 (PMC5711019; doi:10.1371/journal.pone.0188473)
Supplement: S1 Table — (PDF) [file pone.0188473.s001.pdf]

Table 1. Lozano-Velasco et al.

Gene	Specie	Oligo	Sequences
Atp2a2 (NM_001110139.2)	*Rattus novergicus*	Rn_Atp2a2_F	CTGTCCATGTCCCTCCACTT
		Rn_Atp2a2_R	CCTCCAGATAGTTCCGAGCA
Camk2a (NM_012519.2)	*Rattus novergicus*	Rn_Camk2a_F	TGGAAGGGATGGACTTTCAC
		Rn_Camk2a_R	ATTCTGCCACTTCCCATCAC
Cat (NM_012520.2)	*Rattus novergicus*	Rn_Cat_F	GAGAACATTGCCAACCACCT
		Rn_Cat_R	CTTTTCCCTTGGCAGCTATG
Casq2 (NM_017131.2)	*Rattus novergicus*	Rn_Casq2_F	TGGCTATGAGTTCCTGGAGA
		Rn_Casq2_R	CATTCACCACCCCAATCTGT
Enpep (NM_022251.2)	*Rattus novergicus*	Rn_Enpep_F	AAATGCTCAAAGACCCCAAT
		Rn_Enpep_R	CTGCAGCTCAGTGTTGAAGG
Gapdh (NM_017008.4)	*Rattus novergicus*	Rn_Gapdh_F	TCCCATTCTTCCACCTTTGA
		Rn_Gapdh_R	CCAGGGTTTCTTACTCCTTGG
Gpx1 (NM_030826.4)	*Rattus novergicus*	Rn_Gpx1_F	GTCCACCGTGTATGCCTTCT
		Rn_Gpx1_R	GAACTGATTGCACGGGAAAC
Gsr (NM_053906.2)	*Rattus novergicus*	Rn_Gsr_F	GGCCATATCCTAGTGGACGA
		Rn_Gsr_R	AGACCACGGTAGGGATGTTG
GusB (NM_017015.2)	*Rattus novergicus*	Rn_GusB_F	AGATGTACCAGAAGCCAATTATCC
		Rn_GusB_R	ATTCTTTTCGTTTCTCATCCAG
Kcnj2 (NM_017296.1)	*Rattus novergicus*	Rn_Kcnj2_F	TGAAAATGAAGTTGCCCTAACA
		Rn_Kcnj2_R	TCTCCGATTCTCGCCTTAAA
Kcnj12 (NM_053981.2)	*Rattus novergicus*	Rn_Kcnj12_F	GCCACTGACCGAGAAGTGC
		Rn_Kcnj12_R	GGCGCCTGACATGGTAAC
Kcnn3 (NM_019315.2)	*Rattus novergicus*	Rn_Kcnn3_F	ACCAACTGAGGGGTGTCAAG
		Rn_Kcnn3_R	ATTGAAGCTGGCTGTGAGGT
Pitx2 (NM_019334.2)	*Rattus novergicus*	Rn_Pitx2_F	CTGGAAGCCACTTTCCAGAG
		Rn_Pitx2_R	CAGCCCAATTGTTGTACGAG
Pln (NM_022707.2)	*Rattus novergicus*	Rn_Pln_F	GCTGAGCTCCCAGACTTCAC
		Rn_Pln_R	CATGATGCCAGGAAGACAAA
Ryr2 (NM_001191043.1)	*Rattus novergicus*	Rn_Ryr2_F	CTGTGGGATAGGCAACGACT
		Rn_Ryr2_R	GGGAAAAATTCCCAACACCT
Scn1b (NM_001271045.1)	*Rattus novergicus*	Rn_Scn1b_F	TATGGCATCCATCGTGTCAG
		Rn_Scn1b_R	CGCCTGTACAGTTCTCTTTGC
Scn5a (NM_001160162.1)	*Rattus novergicus*	Rn_Scn5a_F	ATGACGGAGGAGCAGAAGAA
		Rn_Scn5a_R	GGAACATGATGGTGACATCG
Sod1 (NM_017050.1)	*Rattus novergicus*	Rn_Sod1_F	CCACTGCAGGACCTCATTTT
		Rn_Sod1_R	CACCTTTGCCCAAGTCATCT
Sod2 (NM_017051.2)	*Rattus novergicus*	Rn_Sod2_F	CCGAGGAGAAGTACCACGAG
		Rn_Sod2_R	GCTTGATAGCCTCCAGCAAC
Wnt8a (NM_001106155.1)	*Rattus novergicus*	Rn_Wnt8a_F	CTGTGGCTGTGATGAGTCAAA
		Rn_Wnt8a_R	CAGCCCTGTTGTTGTGAAGA
Wnt11 (NM_080401.1)	*Rattus novergicus*	Rn_Wnt11_F	CAGGATCCCAAGCCAATAAA
		Rn_Wnt11_R	TAGGCCGGTGTACCACTTTC
Zfhx3 (XM_008772567.2)	*Rattus novergicus*	Rn_Zfhx3_F	CATGCTCAACAACAAGATCCA
		Rn_Zfhx3_R	GGACTCTAGACCTAGACACCGAAA
Cacna1c (NM_001159535.2)	*Mus musculus*	Mm_Cacna1c_F	TCCTGGTCTGAGGAGACGAC
		Mm_Cacna1c_R	GGTGGTGACCTCGATGAACT
Camk2a (NM_177407.4)	*Mus musculus*	Mn_Camk2a_F	AATGGCAGATCGTCCACTTC
		Mm_Camk2a_R	TGGCGACTTCTGTGAACAAG
Cat (NM_009804.2)	*Mus musculus*	Mm_Cat_F	ATCCAGGCTCTTCTGGACAA
		Mm_Cat_R	TCCATCCAGCGTTGATTACA
Gapdh (NM_008084.2)	*Mus musculus*	Mm_Gapdh_F	GGCATTGCTCTCAATGACAA
		Mm_Gapdh_R	TGTGAGGGAGATGCTCAGTG
Gusb (NM_010368.1)	*Mus musculus*	Mm_GusB_F	ACGCATCAGAAGCCGATTAT
		Mm_GusB_R	ACTCTCAGCGGTGACTGGTT

Hcn4 (NM_001081192.1)	*Mus musculus*	Mm_Hcn4_F	CAGCGTCAGAGCGGATACTT
		Mm_Hcn4_R	TGTGGAGGAGGATGGAGTTC
Kcnj2 (NM_008425.4)	*Mus musculus*	Mm_Kcnj2_F	TTGCTTCGGCTCATTCTCTT
		Mm_Kcnj2_F	AGAGATGGATGCTTCCGAGA
Pitx2c (NM_001042502.1)	*Mus musculus*	Mm_Pitx2c_F	CCTCACCCTTCTGTCACCAT
		Mm_Pitx2c_R	GCCCACATCCTCATTCTTTC
Prdx2 (NM_001317385.1)	*Mus musculus*	Mm_Prdx2_F	CTTCGCCAGATCACAGTCAA
		Mm_Prdx2_R	AAATCCAAGCTTCAGGCTCA
Prdx3 (NM_007452.2)	*Mus musculus*	Mm_Prdx3_F	TGGACACCAGAGTCCCCTAC
		Mm_Prdx3_R	TCAAGGCATTGGAAGGATTC
Prdx5 (NM_012021.2)	*Mus musculus*	Mm_Prdx5_F	TGGGAAGGCGACAGACTTAT
		Mm_Prdx5_R	CAGGGCCTCAGAGTTGAGAG
Prdx6 (NM_007453.4)	*Mus musculus*	Mm_Prdx6_F	TTTTGGCCCTGACAAGAAAC
		Mm_Prdx6_R	GAGGGTGGGAACTACCATCA
Ryr2 (NM_023868.2)	*Mus musculus*	Mm_Ryr2_F	TGGGATTGGCAACGATTATT
		Mm_Ryr2_R	GGGAAAAATTCCCAACACCT
Scn5a (NM_021544.4)	*Mus musculus*	Mm_Scn5a_F	CTTCACCAACAGCTGGAACA
		Mm_Scn5a_R	CATGACGAGGAAGAGGAGGA
Sod2 (NM_013671.3)	*Mus musculus*	Mm_Sod2_F	GCAAGGAACAACAGGCCTTA
		Mm_Sod2_R	AGCACCCCAGTCATAGTGCT
Wnt8a (NM_009290.2)	*Mus musculus*	Mm_Wnt8a_F	TTCGTGGACAGTTTGGAGAA
		Mm_Wnt8a_R	GCGGTCATACTTGGCCTTTA
Wnt11 (NM_001285792.1)	*Mus musculus*	Mm_Wnt11_F	GGCCTGTGAAGGACTCAGAA
		Mm_Wnt11_R	ACCACTCTGTCCGTGTAGGG
Zfhx3 (XM_006530585.3)	*Mus musculus*	Mm_Zfhx3_F	GACTGGCAGCTCAACAACAA
		Mm_Zfhx3_R	CCATCAGGTTCGGTTTAGGA
miR-1	*Mus musculus*	hsa-miR-1-3p	UGGAAUGUAAAGAAGUAUGUAU
miR-29a	*Mus musculus*	hsa-miR-29a-3p	UAGCACCAUCUGAAAUCGGUUA
miR-106b	*Mus musculus*	hsa-miR-106b-5p	UAAAGUGCUGACAGUGCAGAU
miR-200a	*Mus musculus*	hsa-miR-200a-3p	UAACACUGUCUGGUAACGAUGU
siEnpep	*Mus musculus*	siEnpep_sense	S 5’: GAUAGUUUAAGACCGAUCA
		siEnpep_asense	AS 3’: CUAUCAAAUUCUGGCUAGU
siSod2	*Mus musculus*	siSod2_sense	S 5’: CUUUCUCAGUAGCGGCAAA
		siSod2_asense	AS 3’: GAAAGAGUCAUCGCCGUUU
siPitx2c	*Mus musculus*	siPitx2c_sense	S 5': GUGCAUACAAUCUCCGAUA
		siPitx2c_asense	AS 3': CACGUAUGUUAGAGGCUAU
