# Supplementary figures and images for: Hyperthyroidism, but not hypertension, impairs PITX2 expression leading to Wnt-microRNA-ion channel remodeling
Source: PLoS One. 2017 Dec 1;12(12):e0188473. doi: 10.1371/journal.pone.0188473 (PMC5711019; doi:10.1371/journal.pone.0188473)

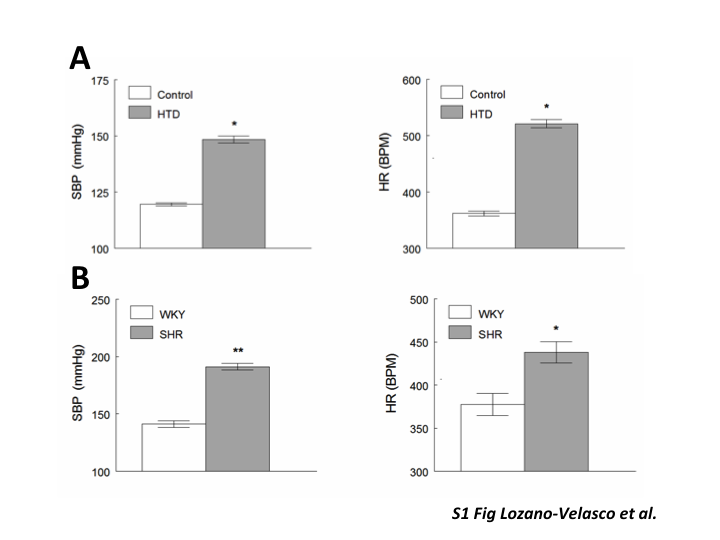

Supplement: S1 Fig — A) Systolic blood pressure (SBP) in mmHg and heart rate (HR) in beats per minute (BPM) measured in control and hyperthyroid (HTD) rats at the end of the experiment (n = 10 each group). Mean ± SEM is displayed. *p<0.001 vs control group. B) Systolic blood pressure (SBP) in mmHg and heart rate (HR) in beats per minute (BPM) measured in Wistar-Kyoto (WKY) and spontaneously hypertensive rats (SHR) at the end of the experiment (n = 10 each group). Mean ± SEM is displayed. *p<0.01, **p<0.001 vs control group. (TIFF) [file pone.0188473.s002.tiff]

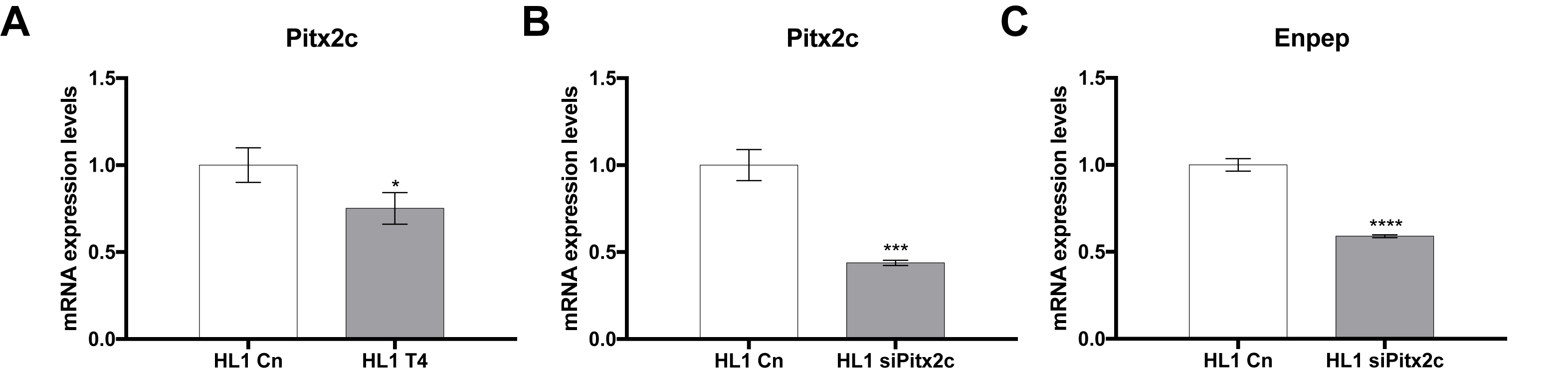

Supplement: S2 Fig — Analyses of Pitx2 expression in primary culture of fetal cardiomyocytes treated with T4 as compared to controls (panel A). Observe that Pitx2 is significantly decreased after T4 administration. Analyses of Pitx2 (panel B) and Enpep (panel C) in HL1 atrial cardiomycytes after Pitx2 siRNA silencing. Observe that Pitx2 siRNA administration significantly decrease Pitx2 expression (panel B) and also Enpep expression (panel C). *p<0.01, ***p<0.001, ****p<0.0001. (TIF) [file pone.0188473.s003.tif]

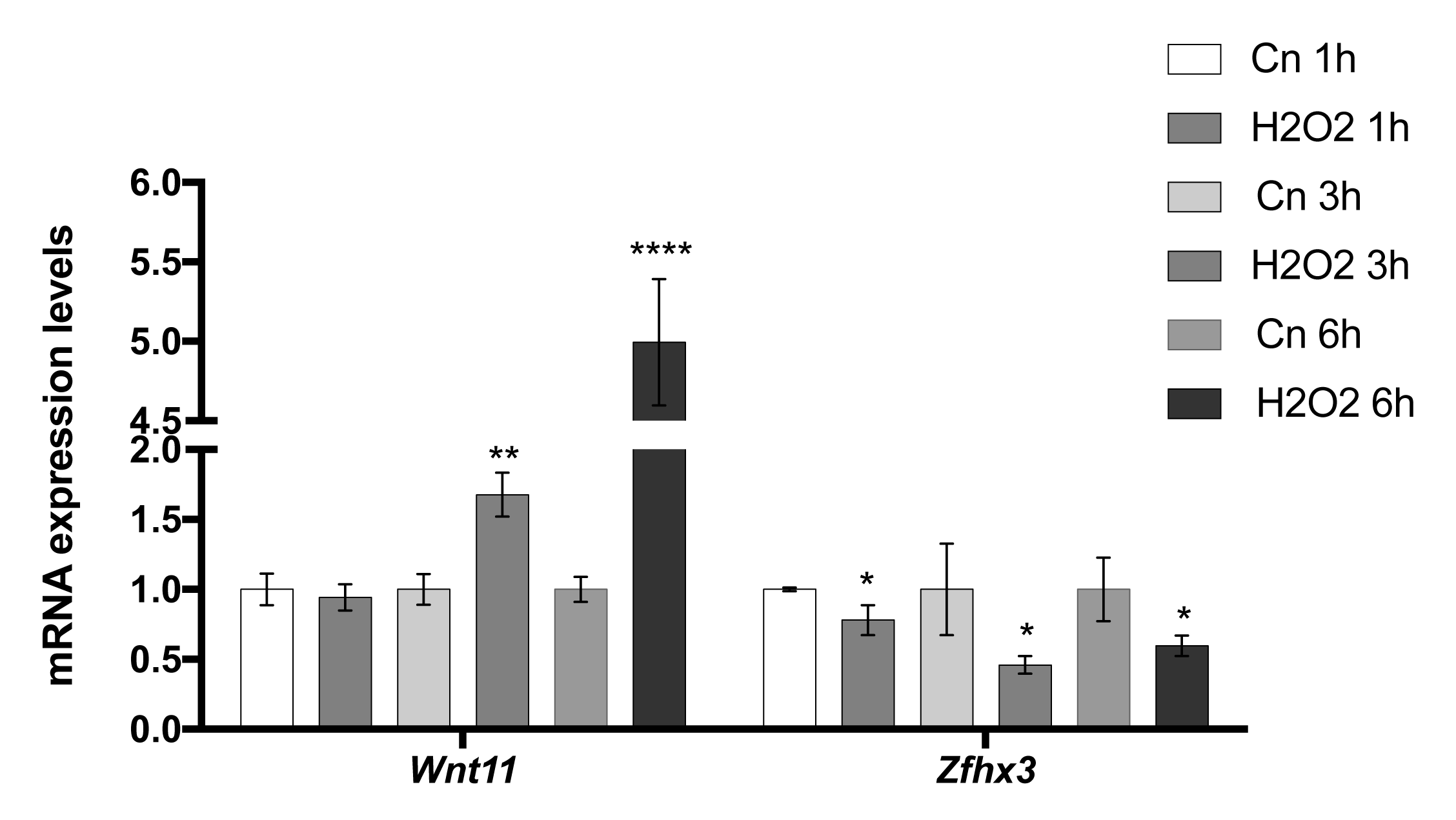

Supplement: S3 Fig — Observe that H202 administration significantly increased Wnt11 at 3h and 6h while significantly decreased Zfhx3 expression at all experimental conditions analyzed. *p<0.01, **p<0.05, ****p<0.0001. (TIF) [file pone.0188473.s004.tif]

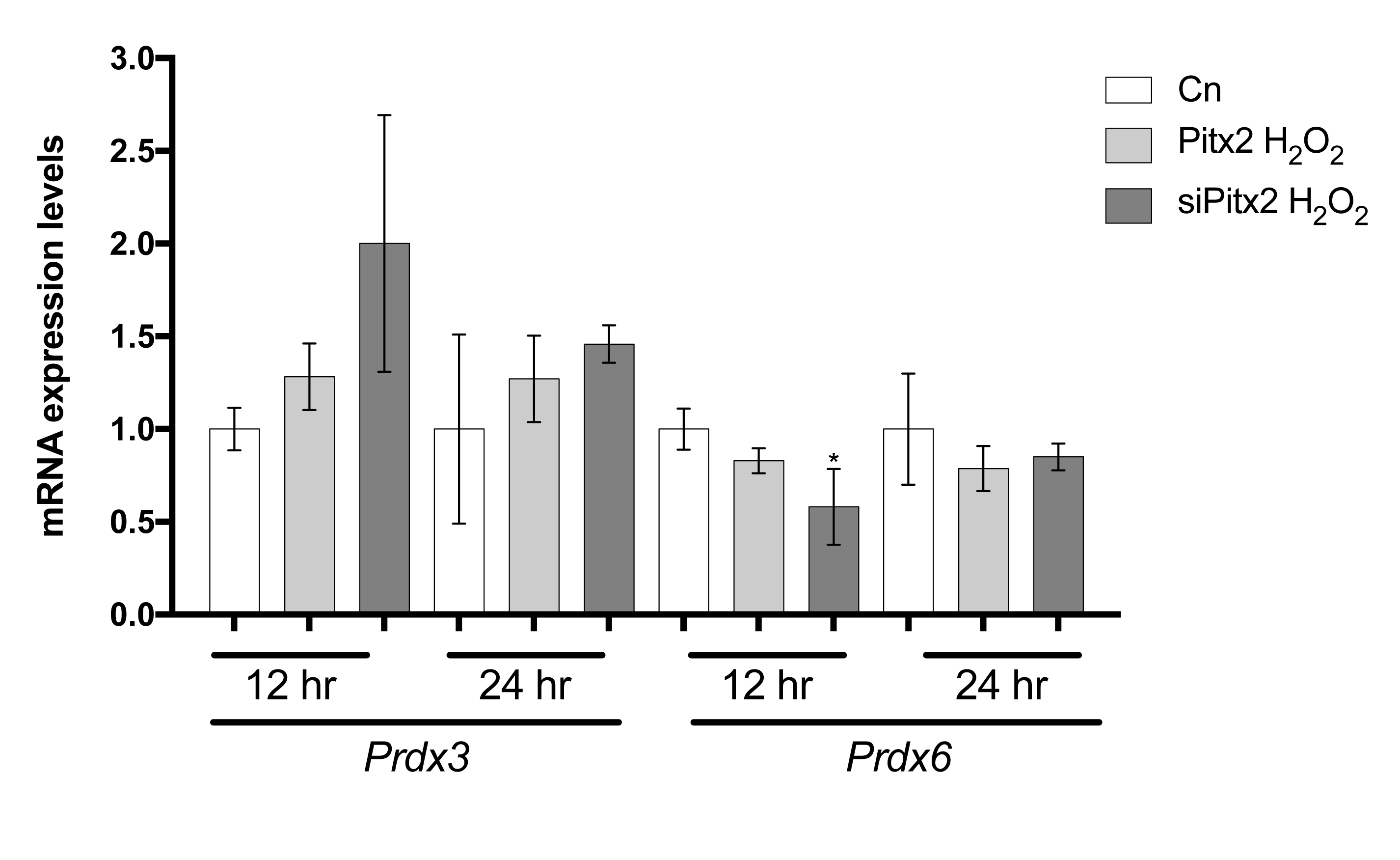

Supplement: S4 Fig — Observe that no significant differences are observed in Prdx3 and Prdx6 expression, except for Prdx6 at 12h after treatment in Pitx2 silencing conditions. *p<0.01. (TIF) [file pone.0188473.s005.tif]
